# 2H,3H-Decafluoropentane-Based Nanodroplets: New Perspectives for Oxygen Delivery to Hypoxic Cutaneous Tissues

**DOI:** 10.1371/journal.pone.0119769

**Published:** 2015-03-17

**Authors:** Mauro Prato, Chiara Magnetto, Jithin Jose, Amina Khadjavi, Federica Cavallo, Elena Quaglino, Alice Panariti, Ilaria Rivolta, Emilio Benintende, Gianfranco Varetto, Monica Argenziano, Adriano Troia, Roberta Cavalli, Caterina Guiot

**Affiliations:** 1 Dipartimento di Neuroscienze, Università di Torino, Torino, Italy; 2 Istituto Nazionale di Ricerca Metrologica (INRIM), Torino, Italy; 3 FujiFilm VisualSonics, Amsterdam, The Netherlands; 4 Dipartimento di Biotecnologie Molecolari e Scienze per la Salute, Molecular Biotechnology Center, Università di Torino, Torino, Italy; 5 Dipartimento di Scienze della Salute, Università di Milano Bicocca, Monza, Italy; 6 Dipartimento di Scienze Chirurgiche, Università di Torino, Torino, Italy; 7 Dipartimento di Scienza e Tecnologia del Farmaco, Università di Torino, Torino, Italy; Center for Molecular Biotechnology, ITALY

## Abstract

Perfluoropentane (PFP)-based oxygen-loaded nanobubbles (OLNBs) were previously proposed as adjuvant therapeutic tools for pathologies of different etiology sharing hypoxia as a common feature, including cancer, infection, and autoimmunity. Here we introduce a new platform of oxygen nanocarriers, based on 2H,3H-decafluoropentane (DFP) as core fluorocarbon. These new nanocarriers have been named oxygen-loaded nanodroplets (OLNDs) since DFP is liquid at body temperature, unlike gaseous PFP. Dextran-shelled OLNDs, available either in liquid or gel formulations, display spherical morphology, ~600 nm diameters, anionic charge, good oxygen carrying capacity, and no toxic effects on human keratinocytes after cell internalization. *In vitro* OLNDs result more effective in releasing oxygen to hypoxic environments than former OLNBs, as demonstrated by analysis through oxymetry. *In vivo*, OLNDs effectively enhance oxy-hemoglobin levels, as emerged from investigation by photoacoustic imaging. Interestingly, ultrasound (US) treatment further improves transdermal oxygen release from OLNDs. Taken together, these data suggest that US-activated, DFP-based OLNDs might be innovative, suitable and cost-effective devices to topically treat hypoxia-associated pathologies of the cutaneous tissues.

## Introduction

Hypoxia is a major feature of several skin pathologies of different etiology, such as bedsores, burns, ulcers, diabetes-associated vasculopathies, methicillin-resistant *Staphilococcus aureus*-infected wounds or even melanomas, all characterized by insufficient oxygen supply to dermal and sub-cutaneous tissues [1–4]. In general, acute and/or mild hypoxia supports adaptation and survival. On the contrary, chronic and/or extreme hypoxia leads to tissue loss. While tumors are metabolically designed to thrive under hypoxic conditions [5], hypoxia in wounds is primarily caused by vascular limitations, further worsened by concomitant conditions (e.g. infection, sympathetic response to pain, hyperthermia, anemia caused by major blood loss, cyanotic heart disease, high altitude) leading to poor healing outcomes [1]. Partial pressure of oxygen in the wound tissue can in principle be increased as adjunct therapy to trigger healing responses and to boost other concomitant therapeutic interventions, e.g. to improve responsiveness to growth factors and acceptance of grafts [6–9]. Currently, hyperbaric oxygen therapy and topical oxygen therapy are practiced [1,10]. Hyperbaric oxygen therapy effectively bolsters tissue oxygen levels and promotes wound healing under specific conditions, however it is expensive, uncomfortable and even dangerous due to fire accident risks [11]. Topical oxygen therapy is cheaper and better accessible for in-home use, but so far inadequately delivers oxygen deep into the skin to fibroblasts, keratinocytes, and inflammatory cells to restore their function [1].

To overcome this problem, intensive research is being pursued to develop new carriers able to release therapeutically significant amounts of oxygen to tissues in an effective and time-sustained manner [12,13]. Hemoglobin (Hb)-based oxygen carriers have been developed as cell-free suspensions, either encapsulated within vehicles, or complexed with protective enzymes [12,13]. Alternative carriers are based on perfluorocarbons, which can carry molecular oxygen without actually binding it, thus favoring gas exchange [14]. However, they are not water-miscible, and therefore need to be formulated into emulsions for *in vivo* use [12,14]. Unfortunately, despite attractive characteristics, no perfluorocarbon-based oxygen emulsion is currently approved for clinical uses: some of them, such as Fluosol-DA, have failed due to secondary effects of the surfactants employed, whereas others, such as Oxygent, displayed adverse cerebrovascular effects on cardiopulmonary bypass [14]. Among the alternative options currently under investigation, perfluorocarbon-based oxygen-loaded microbubbles (OLMBs) have been reported to deliver clinically relevant oxygen amounts in dosages that are approximately 1/500 of the corresponding quantities of other perfluorocarbon-based oxygen carriers [15]. In particular, OLMBs, cored with perfluoropentane (PFP), proved to act as an efficient, biocompatible and stable oxygen delivery system *in vitro* [16]. Formulations were further optimized in order to reach the nanometer size range, and new oxygen-loaded, PFP–cored nanobubbles (OLNBs), both coated with chitosan or dextran, were developed [17,18]. In contrast with the micrometer size, which is generally associated to diagnostic purposes, the nanometer size displays several advantages on a therapeutic level. First, in accordance with Laplace’s law, the smaller the bubble radius, the higher the oxygen partial pressure. Notably, OLNBs remain relatively stable in water for a long time or rise very slowly, gradually shrink, and finally collapse. This in turn leads to reduced diffusivity of OLNBs that helps to maintain adequate kinetic balance of OLNBs against high internal pressure. When needed, oxygen release from OLNBs can be easily promoted upon complementary ultrasound (US) administration. On the contrary, OLMBs tend to increase in size, rapidly rise, and quickly collapse due to long stagnation and dissolution of inner gases into the surrounding water [19]. Therefore, OLNBs appear more clinically feasible to counteract tissue hypoxia than OLMBs. Furthermore, OLNBs are potentially allowed to pass through the nano-sized inter-endothelial gaps of tumor-associated fenestrated capillaries, thus paving the way for potential exploitation in cancer therapy. Finally, nanobubbles have also proven able to carry on molecules other than gaseous oxygen, such as DNA [20], thus suggesting future gene therapy applications [21].

The present work aimed at improving gas delivery to hypoxic tissues by developing a new platform of oxygen nanocarriers based on 2H,3H-decafluoropentane (DFP) and prepared in formulations suitable for topical treatment of dermal tissues. Since DFP is liquid at body temperature, unlike gaseous PFP, these nanocarriers were named oxygen-loaded nanodroplets (OLNDs). While OLNDs keep all the advantages of OLNBs (higher oxygen partial pressure than OLMBs; sensitivity to US and subsequent capability to undergo cavitation events; and ability to pass through the inter-endothelial gaps of fenestrated capillaries), they also display further improvements, appearing more stable and more effective in oxygen storing and releasing, and displaying lower manufacturing costs, no toxicity, and ease of scale-up.

## Materials and Methods

### Materials

Unless otherwise stated, the materials employed here were from Sigma-Aldrich (St Louis, MO).

### Preparation of OLND, OFND, OLNB, OFNB and OSS formulations

#### Preparation of liquid formulations

**Fig 1:**
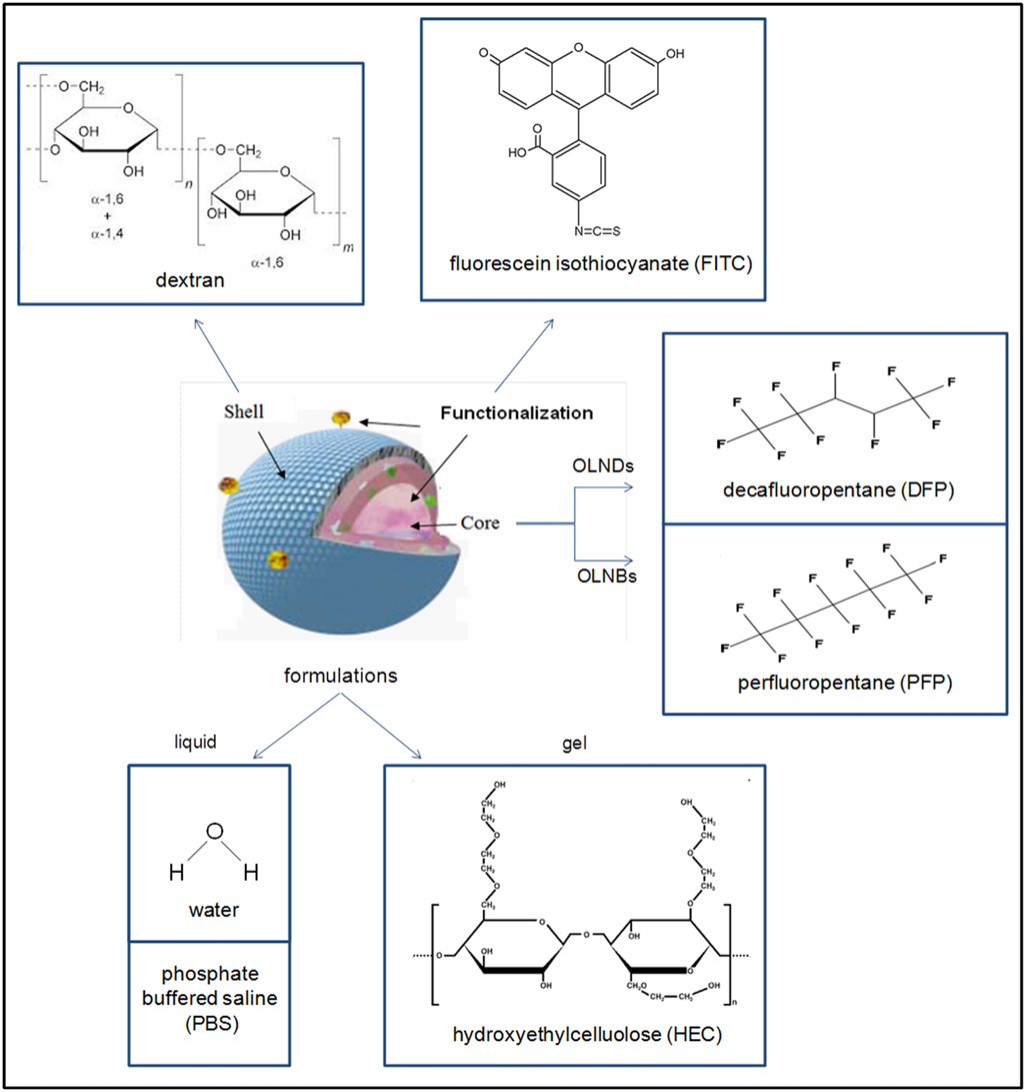
Schematic structure of nanodroplet and nanobubble formulations. The oxygen nanocarriers described in the present work display a core-shell structure. As core fluorocarbon, DFP was employed for OLNDs, whereas PFP was used for OLNBs. Dextran was chosen as polysaccharidic shell molecule for both nanocarriers. In selected experiments, OLNDs were functionalized by conjugation with FITC. All nanocarrier solutions were prepared either in liquid (water or PBS) or gel (2% HEC) formulations.

Compositions and structures of all formulations are detailed in Table 1 and schematized in Fig. 1. For oxygen-loaded nanodroplet liquid formulations, 1.5 ml DFP (Fluka, Buchs, Switzerland) along with 0.5 ml polyvinylpyrrolidone (Fluka, Buchs, Switzerland) and 1.8 ml soy lecithin (Degussa, Hamburg, Germany) solved in 1% w/v ethanol (Carlo Erba, Milan, Italy) and 0.3% w/v palmitic acid solution (Fluka, Buchs, Switzerland) were homogenized in 30 ml water (preparation A) or phosphate buffered saline (PBS) (preparations C-D) for 2 min at 24000 rpm by using Ultra-Turrax SG215 homogenizer (IKA, Staufen, Germany). Ultrapure water was obtained using a 1–800 Millipore system (Molsheim, France). Thereafter, the solution was saturated with O_2_ for 2 min. Finally, 1.5 ml dextran (preparations A, C) or fluorescein isothiocyanate (FITC)-labeled dextran (preparation D) solution was added drop-wise whilst the mixture was homogenized at 13000 rpm for 2 min. For OLNB water formulation, the protocol developed by Cavalli et al. [18] was applied by using PFP as a core fluorocarbon. Oxygen-free nanodroplet (OFND) and nanobubble (OFNB) water formulations were prepared according to OLND and OLNB protocols without adding O_2_. For oxygen-saturated solution (OSS) water formulation, OLND preparation protocol was applied omitting dextran and DFP addition.

**Table 1:** Composition of OLND, OFND, OLNB, OFNB and OSS formulations.

Ingredients	OLNDs				OFNDs		OLNBs		OFNBs		OSS	
	preparation A (%w/v)	preparation B (%w/v)	preparation C (%w/v)	preparation D (%w/v)	preparation A (%w/v)	preparation B (%w/v)	preparation A (%w/v)	preparation B (%w/v)	preparation A (%w/v)	preparation B (%w/v)	preparation A (%w/v)	preparation B (%w/v)
dextran	0.137	0.067	0.137	/	0.137	0.067	0.137	0.067	0.137	0.067	/	/
FITC-dextran	/	/	/	0.137	/	/	/	/	/	/	/	/
DFP	6.868	3.367	6.868	6.868	6.868	3.367	/	/	/	/	/	/
PFP	/	/	/	/	/	/	7.011	3.437	7.011	3.437	/	/
palmitic acid	0.015	0.007	0.015	0.015	0.015	0.007	0.015	0.007	0.015	0.007	0.020	0.010
soy lecithin	0.051	0.025	0.051	0.051	0.051	0.025	0.051	0.025	0.051	0.025	0.060	0.030
polyvinylpyrrolidone	0.070	0.034	0.070	0.070	0.070	0.034	0.070	0.034	0.070	0.034	0.080	0.040
ethanol	3.989	1.956	3.989	3.989	3.989	1.956	3.989	1.956	3.989	1.956	4.400	2.160
filtered H_2_O	88.870	92.584	88.833	88.833	88.870	92.584	88.727	92.514	88.727	92.514	95.440	95.800
NaCl	/	/	0.031	0.031	/	/	/	/	/	/	/	/
sodium phosphate biphasic	/	/	0,006	0,006	/	/	/	/	/	/	/	/
HEC	/	1.960	/	/	/	1.960	/	1.960	/	1.960	/	1.960
O_2_ *	YES	YES	YES	YES	NO	NO	YES	YES	NO	NO	YES	YES

Preparations A: OLND, OFND, OLNB, OFNB and OSS water liquid formulations. Preparations B: OLND, OFND, OLNB, OFNB and OSS 2% HEC gel formulations. Preparation C: OLND PBS liquid formulation. Preparation D: FITC-labeled OLND PBS liquid formulation.

* O_2_ is merely indicated for its presence/absence in the solution (YES/NO), as it was added in excess to reach saturation; specific O_2_ content was further measured during characterization, as shown in Table 2

**Table 2:** Physical-chemical characterization of OLNDs, OFNDs, OLNBs, OFNBs and OSS.

	Inner core fluorocarbon	Fluorocarbon boiling point	Outer shell polysaccharyde	O_2_ content (mg/ml±SD)		diameters (nm±SD)	polidispersity index	zeta potential (mV±SD)
				before UV	after UV			
OLNDs	DFP	51°C	dextran	0.43 ± 0.01	0.42 ± 0.01	596.35 ± 194.09	0.13	-25.68 ± 1.00
OFNDs	DFP	51°C	dextran	/	/	239.54 ± 96.20	0.10	-25.17 ± 1.00
OLNBs	PFP	32°C	dextran	0.43 ± 0.01	0.42 ± 0.01	486.87 ± 147.62	0.11	-27.31 ± 1.00
OFNBs	PFP	32°C	dextran	/	/	212.3100B0031 94.82	0.95	-26.54 ± 1.00
OSS	/	/	/	0.41 ± 0.01	0.40 ± 0.01	/	/	/

**Fig 2:**
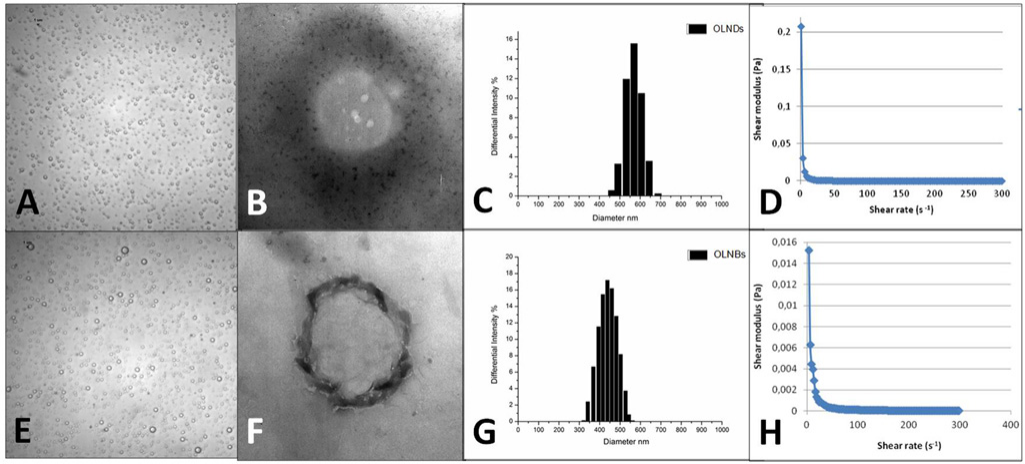
OLND and OLNB morphology, size distribution, and shell shear modulus. OLNDs and OLNBs were checked for morphology by optical microscopy or by TEM, for size distribution by light scattering, and for shell shear modulus by rheometry. Results are shown as representative images from ten different preparations. Panel A. OLND image by optical microscopy. Magnification: 60X. Panel B. OLND image by TEM. Magnification: 21000X. Panel C. OLND size distribution. Panel D. OLND flow curve. Panel E. OLNB image by optical microscopy. Magnification: 60X. Panel F. OLNB image by TEM. Magnification: 21000X. Panel G. OLNB size distribution. Panel H. OLNB flow curve.

**Fig 3:**
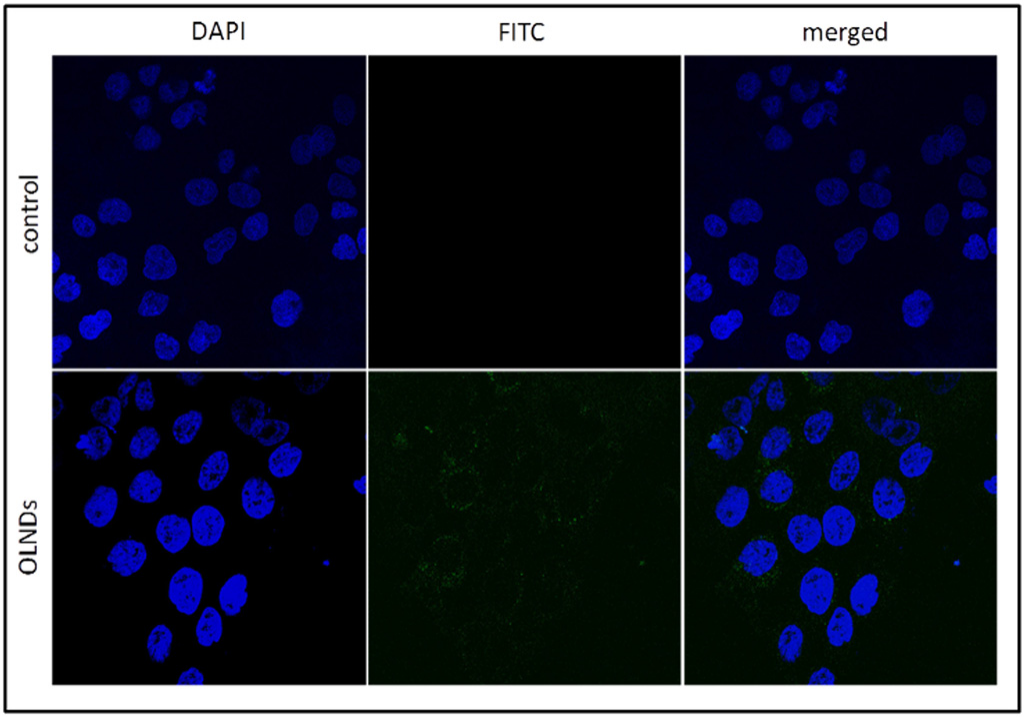
OLND internalization by HaCaT cell line. Human keratinocytes (10^6^ cells /2 ml Panserin medium) were left untreated (upper panels) or treated with 200 l FITC-conjugated OLND PBS formulation (lower panels) for 24 h in normoxia (20% O_2_). After DAPI staining, cells were checked by confocal microscopy. Results are shown as representative images from three independent experiments. Left panels: cell nuclei after DAPI staining, in blue. Central panels: FITC-conjugated OLNDs, in green). Right panels: merged images. Magnification: 63X.

Liquid formulations were characterized for average diameters, polydispersity index, and zeta potential by light scattering, and for oxygen content through a chemical assay (see Materials and Methods). Results are shown as means ± SD from ten preparations (average diameters, polydispersity index, and zeta potential) or three preparations (oxygen content) for each formulation. See also Figs. 1–3 and Table 1 for further detail on OLND or OLNB structure, morphology, size distribution, and formulations.

#### Preparation of gel formulations

To obtain gel formulations (preparations B, Table 1), 0.8 mg hydroxyethylcellulose were solved in 20 ml water, and subsequently mixed 1:1 with OLND, OFND, OLNB, OFNB, or OSS water formulations.

#### Sterilization

OLNDs, OFNDs, OLNBs, OFNBs, and OSS were sterilized through UV-C exposure for 20 min. Thereafter, UV-C-treated materials were incubated with cell culture RPMI 1640 medium (Invitrogen, Carlsbad, CA) in a humidified CO_2_/air-incubator at 37°C up to 72 h, not displaying any signs of microbial contamination when checked by optical microscopy. Moreover, UV-C-sterilized O_2_-containing solutions underwent further analyses through O_3_ measurement and electron paramagnetic resonance (EPR) spectroscopy (Miniscope 100 EPR spectrometer, Magnettech, Berlin, Germany), showing no O_3_ generation and negligible singlet oxygen levels immediately after UV-C exposure.

### Characterization of nanodroplets and nanobubbles

#### Morphology, average diameters, and shell thickness

The morphology of nanodroplet and nanobubble formulations was determined by transmitting electron microscopy (TEM) and by optical microscopy. TEM analysis was carried out using a Philips CM10 instrument (Philips, Eindhoven, The Netherlands), whereas optical microscopy was carried out using a XDS-3FL microscope (Optika, Ponteranica, Italy). For TEM analysis nanodroplet and nanobubble formulations were dropped onto a Formvar-coated copper grid (Polysciences Europe GmbH, Eppelheim, Germany) and air-dried before observation. Furthermore, average diameters and shell thickness were elaborated from TEM images.

#### Size, particle size distribution, zeta potential, refractive index, viscosity, and shell shear modulus

Sizes, polydispersity indexes, and zeta potentials of nanodroplets and nanobubbles were determined by dynamic light scattering using Delsa Nano C instrument (Beckman Coulter, Brea, CA), displaying a (0.6 nm—7 μm) range for measurements of particle size distribution. Each value reported is the average of three measurements of ten different formulations. The polydispersity index indicates the size distribution within a nanodroplet or nanobubble population. For zeta potential determination, formulation samples were placed into an electrophoretic cell, where an electric field of approximately 30 V/cm was applied. Each sample was analyzed at least in triplicate. The electrophoretic mobility was converted into zeta potential using the Smoluchowski equation [22]. The refractive indexes of OLND and OLNB formulations were calculated through a polarizing microscope (Spencer Lens Company, Buffalo, New York). The viscosity and the shell shear modulus were determined through Discovery HR1 Hybrid Rheometer (TA instruments, Milan, Italy).

#### Oxygen content

Immediately after preparation, oxygen content of OLNDs, OLNBs and OSS was evaluated for characterization purposes by adding known amounts of sodium sulfite and measuring generated sodium sulfate, according to the reaction:

$$Na_{2}SO_{3}+O_{2}\to Na_{2}SO_{4}$$

Afterwards, during all the subsequent experiments, an oxymeter was employed.

#### Stability

The stability of formulations stored at 4°C, 25°C or 37°C was evaluated over time up to 6 months by determining morphology, sizes and zeta potential of nanodroplets and nanobubbles by optical microscopy and light scattering.

### Biocompatibility assessment

#### Human keratinocyte cell cultures

HaCaT (Cell Line Service GmbH, Eppelheim, Germany), a long-term cell line of human keratinocytes immortalized from a 62-year old Caucasian male donor [23], was used for assessment of OLND biocompatibility. Cells were grown as adherent monolayers in Dulbecco’s modified Eagle’s medium supplemented with 10% fetal bovine serum, 100 U/ml penicillin, 100 μg/ml streptomycin (Cambrex Bio Science, Vervies, Belgium) and 2 mM L-glutamine in a humidified CO_2_/air-incubator (Thermo Fisher Scientific Inc., Waltham, MA) at 37°C. Before starting the experiments, cells were washed with PBS, detached with trypsin/ethylenediaminetetraacetic acid (0.05/0.02% v/v), washed with fresh medium and plated at a standard density (10^6^ cells/well in 6-well plates) in 2 ml fetal bovine serum-free Panserin 601 medium (PAN Biotech, Aidenbach, Germany) to prevent serum interference in the toxicity assay.

#### Evaluation of OLND uptake by human keratinocytes

HaCaT cells were plated in 24-well plates on glass coverslips and incubated in Panserin 601 medium for 24 h with/without 200 μl FITC-labeled OLNDs in a humidified CO_2_/air-incubator at 37°C. After 4',6-diamidino-2-phenylindole (DAPI) staining to visualize cells nuclei, fluorescence images were acquired by a LSM710 inverted confocal laser scanning microscope (Carl Zeiss, Oberkochen, Germany) equipped with a Plan-Neofluar 63×1.4 oil objective, that allowed a field view of at least 5 cells. Wavelength of 488 nm was used to detect OLNDs, and of 460 nm to detect the labeled nuclei. The acquisition time was 400 ms.

#### OLND cytotoxicity

The potential cytotoxic effects of OLNDs were measured as the release of lactate dehydrogenase (LDH) from HaCaT cells into the extracellular medium. Briefly, cells were incubated in Panserin 601 medium for 24 h in the presence or absence of increasing doses (100–400 μl) of OLNDs, either in normoxic (20% O_2_) or hypoxic (1% O_2_) conditions, in a humidified CO_2_/air-incubator at 37°C. Then, 1 ml of cell supernatants was collected and centrifuged at 13000*g* for 2 min. Cells were washed with fresh medium, detached with trypsin/ethylenediaminetetraacetic acid (0.05/0.02% v/v), washed with PBS, resuspended in 1 ml of TRAP (82.3 mM triethanolamine, pH 7.6), and sonicated on ice with a 10 s burst. 5 μl of cell lysates and 50 μl of cell supernatants were diluted with TRAP and supplemented with 0.5 mM sodium pyruvate and 0.25 mM NADH (300 μL as a final volume) to start the reaction. The reaction was followed measuring the absorbance at 340 nm (37°C) with Synergy HT microplate reader (Bio-Tek Instruments, Winooski, VT). Both intracellular and extracellular enzyme activities were expressed as μmol of oxidized NADH/min/well. Finally, cytotoxicity was calculated as the net ratio between extracellular and total (intracellular + extracellular) LDH activities.

#### Human keratinocyte cell viability

Cell viability was evaluated using 3-(4,5-dimethylthiazol-2-yl)-2,5-diphenyltetrazolium bromide (MTT) assay. HaCaT cells were incubated in Panserin 601 medium for 24 h with/without increasing doses (100–400 μl) of OLNDs, either in normoxic (20% O_2_) or hypoxic (1% O_2_) conditions, in a humidified CO_2_/air-incubator at 37°C. Thereafter, 20 μL of 5 mg/mL MTT in PBS were added to cells for 3 additional hours at 37°C. The plates were then centrifuged, the supernatants discarded and the dark blue formazan crystals dissolved using 100 μL of lysis buffer containing 20% (w/v) sodium dodecyl sulfate, 40% N,N-dimethylformamide (pH 4.7 in 80% acetic acid). The plates were then read on Synergy HT microplate reader at a test wavelength of 550 nm and at a reference wavelength of 650 nm.

### *In vitro* determination of oxygen release from OLNDs

#### Oxygen release without ultrasound

The concentration of oxygen released by diffusion from OLND, OLNB and OSS liquid or gel formulations into a hypoxic solution was monitored up to 6 h through Hach Lange LDO oxymeter (Hach Lange, Derio, Spain), displaying an accuracy of 0.01 mg/l. Before each measurement, the oxymeter was calibrated in air, waiting for stable temperature and humidity conditions to be reached.

#### Oxygen release with ultrasound and trespassing of skin membranes

To study the ability of US-activated OLND and control formulations to release O_2_ through biological membranes, a US probe with a high frequency transducer (*f* = 2.5 MHz; *P* = 5 W) was used, combined with a home-made apparatus with two sealed cylindrical chambers (lower chamber: OLND, OFND, OLNB, OFNB or OSS solutions; upper chamber: hypoxic solution) separated by a layer of pig ear skin employed as a model of biological membrane, as previously described [24]. The US transducer (*f* = 2.5 MHz; *P* = 5 W) was alternatively switched on and off at regular time intervals of 5 min for an overall observational period of 135 min, and oxygen concentration in the recipient chamber was monitored by Hach Lange LDO oxymeter every 45 min. The probe was employed in continuous mode. The wave was sinusoidal. No inertial cavitation was observed at applied settings. Because of the local heating caused by US, the O_2_ sensor was positioned laterally in order to prevent possible damage of the oxymeter, whereas the transducer was held in a fixed position, within the donor compartment. The acoustic power of the transducer was determined through a balance's radiation force with a reflecting target, with an uncertainty of 4%. Of note, LDO Hach Lange oxymeter also allowed to measure the temperature of the solution, which never exceeded 30°C in our experiments.

### *In vivo* determination of oxygen release from OLNDs

#### Mice

BALB/c mice were bred under specific pathogen-free conditions by Fujifilm Visualsonics (Amsterdam, The Netherlands) or at the Molecular Biotechnology Center (Torino, Italy). Before performing the experiments, healthy mice were shaved locally (abdomens or hind limbs depending on the study, as described in the following paragraphs) and anesthetized by injecting intramuscularly a mixture of tiletamine/zolazepam 20 mg/Kg (Zoletil 100, Carros Cedex, France) and 5 mg/Kg xylazine (Rompun, Bayer, Leverkusen, Germany). All procedures were done in accordance with the EU guidelines and with the approval of the Università di Torino animal care committee (16/03/2011).

#### Measurement of oxy/deoxy-Hb levels without ultrasound (photoacoustic imaging)

The shaved hind limbs of nine anesthetized mice were topically treated with OLND, OFND or OSS gel formulations. Before, during and after treatment (10 min), the subcutaneous levels of oxy- and deoxy-Hb were monitored through Vevo LAZR photoacoustic imager (Fujifilm Visualsonics, Amsterdam, the Netherlands) featuring a hybrid US transducer (central *f =* 21 MHz; spatial resolution = 75 μm).

#### Measurement of tcpO_2_ with ultrasound

The shaved abdomens of eight anesthetized mice were topically treated with OLNDs and sonicated using a home-made US equipment (*f* = 1 MHz; P = 5 W; t = 30 sec). US probe was employed at f = 1 MHz since this is the frequency value routinely employed in clinical practice. Temperature changes after 30 sec US administration were neglectable. Before and after treatment (1 h), the transcutaneous tension of oxygen (*tcpO*_*2*_) was measured through TINA TCM30 oxymeter (Radiometer, Copenhagen, Denmark). All *tcpO*_*2*_ measurements were taken after physiological stabilization.

### Statistical analysis

Every characterization of ten preparations for each formulation was performed in triplicate, and results are shown as means ± SD (light scattering and oxygen measurement) or as a representative image (TEM and optical microscopy, rheological analysis). Data from cell studies are shown as means ± SEM (LDH and MTT) or as a representative image (confocal microscopy) from three independent experiments analyzed in duplicate. Results from *in vitro* oxygen release studies are shown as a representative image (release without US) or as means ± SD (release with US) from three independent experiments. Results from *in vivo* oxygen release studies are shown as a representative image (release without US) or as means ± SD (release with US) from eight mice. SD or SEM were used for descriptive or inferential information, respectively (see Cumming et al [25] for an exhaustive review). Data were analyzed for significance by Student’s *t* test (software: Fig.P for Windows, Fig.P Corporation, Hamilton, ON, Canada) or by a one-way Analysis of Variance (ANOVA) followed by Tukey's post-hoc test (software: SPSS 16.0 for Windows, SPSS Inc., Chicago, IL).

## Results

### Characterization of OLND and control formulations

After manufacturing OLNDs and control preparations were characterized for: morphology and shell thickness, by optical microscopy and TEM; size, particle size distribution, polydispersity index and zeta potential, by dynamic light scattering; refractive index by polarizing microscopy; viscosity and shell shear modulus by rheometry; and oxygen content (before and after UV-C sterilization) through a chemical assay. Results are shown in Fig. 2 and Tables 2–3. Both nanodroplets and nanobubbles displayed spherical shapes. All sizes were in the nanometer range, with average diameters ranging from ~490 nm (OLNBs) to ~590 nm (OLNDs) for oxygen-loaded carriers and from ~210 nm (OFNBs) to ~240 nm (OFNDs) for oxygen-free carriers. All diameters were hydrodynamic, sphere equivalent, and were volume weighted. Zeta potentials ranged from ~ –27 to ~ –25 mV. Refractive indexes were similar for both OLND and OLNB formulations (~ 1.33). OLND formulation displayed a viscosity value of 1.59188 e-3 Pa·s and a shear modulus value of 5.43 e-2 mPa, calculated at a shear rate value of 150 s^-1^. OLNB formulation displayed a viscosity value of 1.94426 e-3 Pa·s and a shear modulus value of 6 e-2 mPa mPa, calculated at a shear rate value of 150 s^-1^. OLNDs displayed a good oxygen-storing capacity of about 0.40 mg/ml of oxygen before and after 20-min UV-C sterilization. Such oxygen amount was comparable with that of OLNBs or OSS, thus similar volumes of OLND, OLNB and OSS preparations were further employed in the subsequent experiments.

**Table 3:** Refractive indexes of OLND and control formulations.

Formulation	Refractive index
water	1.332 ± 1
1% dextran solution	1.333 ± 1
OLNDs	1.336 ± 1
OLNBs	1.335 ± 1

Water, 1% dextran solution, OLND, and OLNB formulations were analyzed for refractive indexes through polarizing microscopy. Results are shown as means ± SD from three preparations for each formulation.

### OLND biocompatibility

OLND biocompatibility with human cells was evaluated by testing *in vitro* cultures of human HaCaT keratinocytes (10^6^ cells/2 ml Panserin 601 medium). As firstly checked by confocal microscopy (Fig. 3), HaCaT cells avidly internalized FITC-conjugated OLNDs.

**Fig 4:**
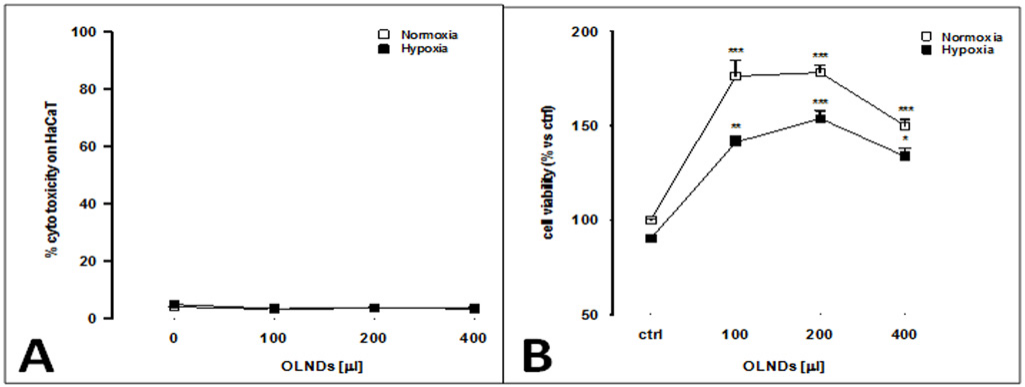
OLND are not cytotoxic and improve viability of human keratinocytes *in vitro*. Human keratinocytes (10^6^ cells/2 ml Panserin 601 medium) were left untreated or treated with different doses (100–400 l) of OLND PBS formulation for 24 h in normoxia (20% O_2_; white-squared curves, both panels) or hypoxia (1% O_2_; black-squared curves, both panels). Thereafter, OLND cytotoxicity (Panel A) was measured through LDH assay, whereas cell viability (Panel B) was measured through MTT assay. Results are shown as means ± SEM from three independent experiments. Data were also evaluated for significance by ANOVA. Panel A. Versus normoxic untreated cells: *p* not significant. Panel B. Versus normoxic untreated cells: * *p* < 0.01; ** *p* < 0.001; *** *p* < 0.0001.

Thereafter, OLND toxicity and cell viability both in normoxic (20% O_2_) and hypoxic (1% O_2_) conditions were evaluated by LDH and MTT assays, respectively. As shown in Fig. 4A, increasing volumes of OLND PBS suspensions, ranging from 100 to 400 μl, did not result toxic to cells either in normoxia or hypoxia. Eventually, OLNDs improved keratinocyte viability in either condition of oxygenation (Fig. 4B).

### *In vitro* oxygen release from OLNDs

**Fig 5:**
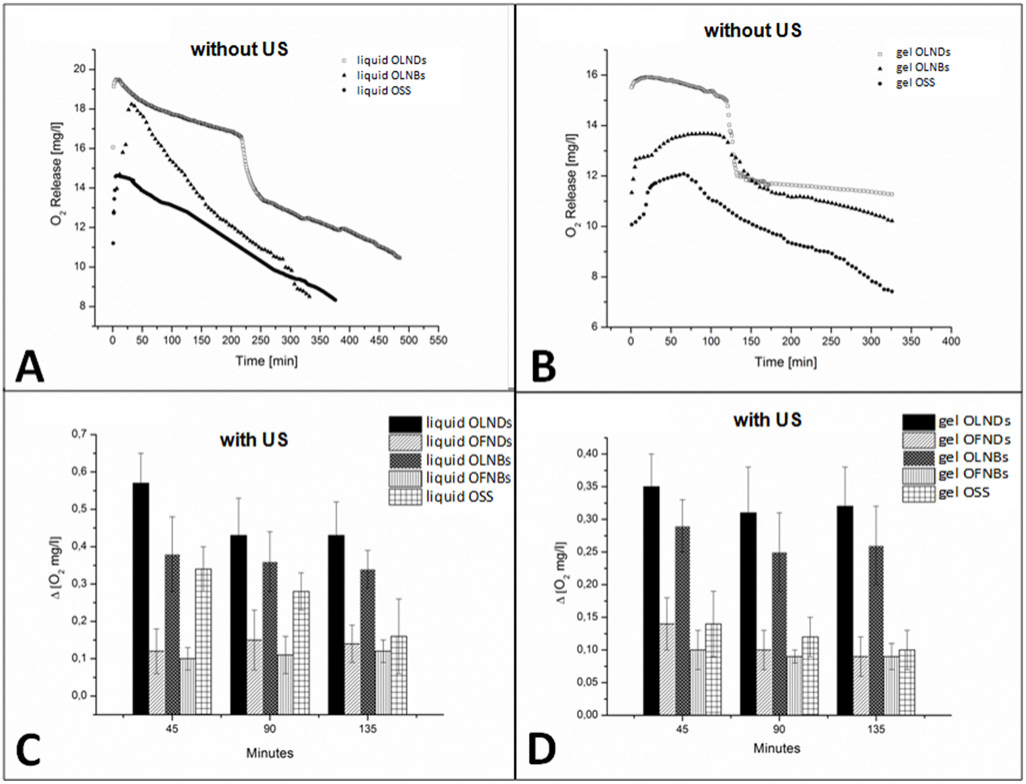
*In vitro* oxygen release from OLND liquid and gel formulation and US-triggered sonophoresis through skin membranes. Panels A-B. Oxygen release without US. OLND, OLNB and OSS water (A) and 2% HEC gel (B) formulations were monitored up to 6 h through an oxymeter for oxygen delivery by diffusion. Results are shown as a representative image from three independent experiments. Panels C-D. Oxygen release with US. US abilities to induce sonophoresis and oxygen release from OLND and control water (C) or 2% HEC gel (D) formulations were evaluated up to 135 min. Changes in oxygen levels in the hypoxic chamber between each time interval (0–45 min; 45–90 min; and 90–135 min) are indicated as ΔO_2_. Results are shown as means ± SD from three independent experiments. Data were also evaluated for significance by ANOVA. Versus OLND formulation: *p* < 0.001.

OLND, OLNB, and OSS abilities to release oxygen *in vitro* were comparatively evaluated. Following dissolution of the same amount of nanocarrier-containing water or 2% HEC gel formulatios in a hypoxic solution, OLNDs released larger amounts of oxygen for longer times (up to 6 h) than OLNBs and OSS (Fig. 5A-B). Of note, the dynamics of oxygen release from OLNDs over time was characterized by two subsequent phases: first, OLNDs tended to agglomerate, thus leading to a plateau in the graph; then, they simultaneously delivered oxygen, thus leading to a great dip-off in the graph. Oxygen release was also compared following sonication. As shown in Fig. 5C-D, US (*f* = 2.5 MHz; P = 5 W) improved the ability of OLNDs to cross the pig skin membrane and to release oxygen into the hypoxic chamber up to 135 min, and such oxygen release was larger than that from OFND, OLNB, OFNB, and OSS liquid or gel formulations.

### *In vivo* oxygen release from OLNDs

**Fig 6:**
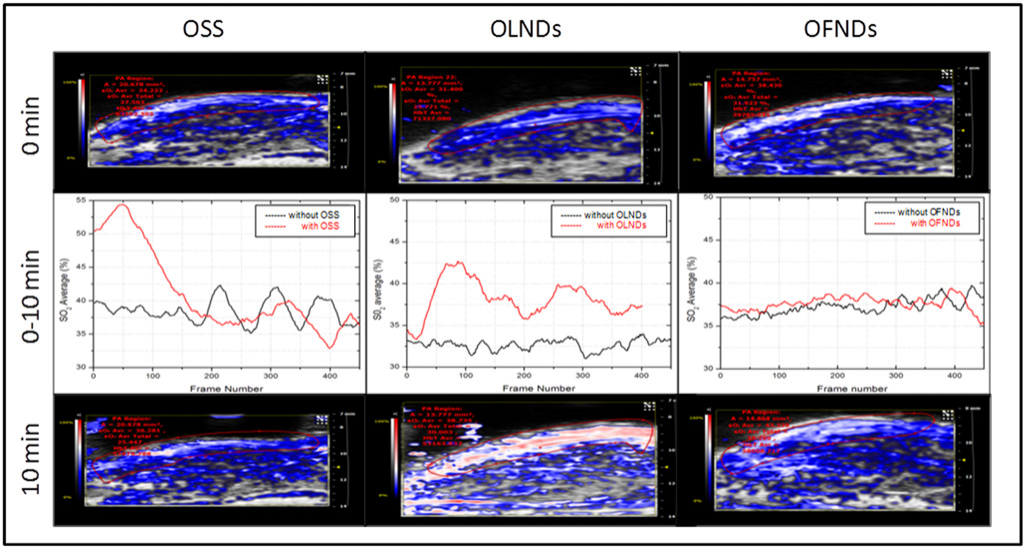
Topical treatment with OLND gel formulation effectively enhances oxy-Hb levels *in vivo*. The shaved hind limbs of nine anesthetized mice were monitored by photoacoustics for oxy-Hb and deoxy-Hb levels before (0 min, upper row), during (0–10 min, central row) and after (10 min, lower row) topical treatment with OSS (first column), OLND (second column) and OFND (third column) gel formulations. White/red pixels: oxy-Hb; blue pixels: deoxy-Hb. Data are shown as representative images from three independent experiments (three mice per experiment) with similar results.

The skin oxygenation of the shaved hind limbs of nine anesthetized mice topically treated with OLND, OFND or OSS gel formulations was monitored by visualizing the subcutaneous levels of oxy-Hb and deoxy-Hb through photoacoustic imaging before, during and after the treatment (t = 10 min). As shown in Fig. 6, oxy-Hb levels significantly increased for the entire observational period in the animals treated with OLNDs. As expected, OSS induced a high but only transient peak in oxy-Hb, whereas OFNDs did not affect oxy-/deoxy-Hb balances at all.

**Fig 7:**
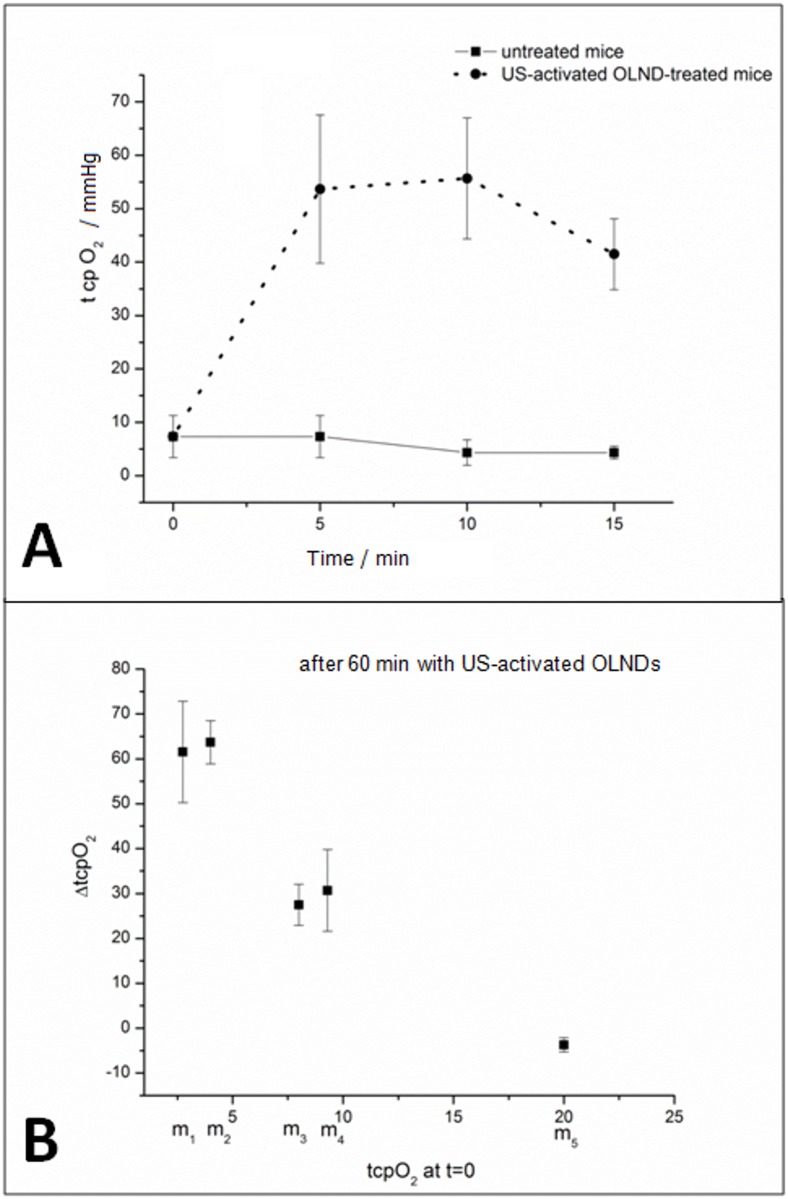
Topical treatment with US-activated OLNDs effectively enhances *tcpO*_*2*_ *in vivo*. The shaved abdomens of eight anesthetized mice were topically treated with OLND gel formulation and sonicated (*f* = 1 MHz; P = 5 W; t = 30 sec). Before and after treatment, *tcpO*_*2*_ was monitored through transcutaneous oxymetry. Panel A. Short-term time-course (0–15 min) *tcpO*_*2*_ monitoring of three mice before and after treatment with OLND gel formulation. Data are shown as means ± SD. Results were also analyzed for statistical significance by Student’s *t* test. Versus untreated mice: *p* < 0.01. B. Long-term end-point (1 h) *tcpO*_*2*_ measurement of five mice (m1-m5) before and after treatment with OLND gel formulation. Data are shown individually per each mouse.

Thereafter, OLND ability to improve tissue oxygenation *in vivo* upon US treatment was investigated. The shaved abdomens of eight anesthetized mice were topically treated with OLNDs and sonicated for 30 sec (*f* = 1 MHz; P = 5 W). Skin oxygenation was investigated through transcutaneous oxymetry (Fig. 7: panel A, 0–15 min; panel B, 1 h) before and after the treatment. Basal *tcpO*_*2*_ values in mice were inhomogeneous, possibly as a consequence of the different level of peripheral vasoconstriction induced by anesthesia. Nevertheless, after topical administration of US-activated OLNDs hypoxic mice displayed larger oxygenation levels in a time-sustained manner up to 1 h.

## Discussion

The major novelty of the present nanocarriers (OLNDs) is the oxygen-storing core structure consisting in DFP. Notably, DFP displays oxygen-solubilizing capabilities as well as PFP [24, 26–27], the most widely used fluorocarbon for oxygenating emulsions and nanobubble formulations [12, 14, 17–18]. However, in DFP the carbon skeleton is surrounded by ten fluorine and two hydrogen atoms, whereas in PFP it is surrounded by twelve fluorine atoms (see Fig. 1). Therefore, while binding oxygen molecules, DFP can also establish hydrogen bonds between hydrogen and oxygen atoms in addition to instantaneous dipoles between fluorine and oxygen atoms (as for PFP). The hydrogen bond (5 to 30 kJ/mole) is stronger than a van der Waals interaction, but weaker than covalent or ionic bonds. As such, the presence of DFP in the core makes OLNDs more stable than OLNBs, without compromising their ability to release oxygen. However, unlike PFP, which has a boiling point of 32°C and is gaseous at body temperature, DFP boils at 51°C and is therefore liquid at 37°C. For this reason, the new nanocarriers are actually nanodroplets. On the other hand, dextran was kept as the main constituent of the polysaccharidic shell as for former OLNBs [18], since dextran-based formulations have been extensively tested for biocompatibility [28–29], and dextran-based hydrogels are currently used as matrices in tissue engineering, without showing signs of inflammation *in vivo* [30]. Recent toxicological studies on mechanically processed polysaccharides of different molecular weight showed that dextran, along with the products of its mechano-chemical processing, can be classified as class 4 (low-toxicity) substance [31].

OLND properties were challenged by comparison with several control preparations including OLNBs, OFNDs, OFNBs, and OSS. All preparations were manufactured either in liquid (water or PBS solution) or gel (2% w/v HEC) formulations. Following nanocarrier manufacturing, UV-C exposure for 20 min was chosen as a sterilizing procedure, which proved to be effective without significantly inducing O_3_ or singlet oxygen generation.

OLNDs and control preparations were characterized for morphology, average diameters, shell thickness, particle size distribution, polydispersity index, zeta potential, refractive index, viscosity, shell shear modulus, and oxygen content (before and after UV sterilization). Both OLNDs and OLNBs displayed spherical shapes, nanometric sizes, similar viscosity and shell shear modulus, and negative charges. Interestingly, the diameters of OLNDs and OLNBs resulted increased by almost three times with respect to the oxygen-free formulations. Such an increase can be related to the different solubility of oxygen either in DFP or PFP. Indeed, the presence of oxygen in the core of the formulations can change the interfacial layer structure, modify the surface tension, and lead to different hydrophobicity [26–27]. This might also explain the slightly larger size of OLNDs with respect to OLNBs. On the other hand, OLND and OLNB negative charges are a likely consequence of the presence of dextran in the outer shell. Indeed, although dextran is a neutral polymer, it is well known that when it is immerged in a saline solution (such as PBS here) it acquires negative polarity [32]. Besides, the zeta potential measures charge repulsion or attraction between particles. Therefore, it is also a fundamental parameter to determine nanoparticle physical stability, with zeta potentials lower than-30 mV or larger than +30 mV being generally required for physical stability of colloid systems [22]. Although nanodroplets and nanobubbles displayed zeta potentials slightly larger than-30 mV, our formulations proved to be physically stable over time for the steric repulsion of the polymer chains, as assessed by monitoring their sizes and zeta potential by dynamic light scattering up to 6 months after manufacturing. In addition, nanoparticle charge makes them suitable for topical treatment, enhancing their interaction with skin and improving their therapeutic effect on inflamed cutaneous tissues, either without [33] or with concomitant US treatment [34]. Interestingly, although cationic nanoparticles are generally preferred for topical treatment due to the anionic nature of the skin [35], some authors have shown that anionic nanoparticles can be more effective [36] and less toxic [37] than the cationic ones.

Furthermore, OLND solution displayed a good oxygen-storing capacity (0.4 mg O_2_/ml) either before or after UV-C sterilization. The partial pressure of oxygen (pO2) in dermal wounds ranges from 0 to 10 mm Hg centrally to 60 mm Hg at the periphery, whereas the pO2 in the arterial blood is approximately 100 mm Hg [38]. The rate of oxygen consumption in the wound is determined by the availability of oxygen as a substrate and the local metabolic conditions in the wound. This drives home the rationale for providing supplemental oxygen to wounds and explains why wounds that are profoundly ischemic (*tcpO*_*2*_ < 20 mm Hg) fail to heal and are more prone to infection [38]. Interestingly, Davis and colleagues have demonstrated using a pig model that a topically applied perfluorocarbon emulsion with an oxygen concentration of 2 mL O_2_/ml can increase the rate of wound epithelialization, thus providing supporting evidence from a preclinical model that supplementing oxygen delivery using a topical approach can improve healing outcomes [39].

OLND toxicity on human cells was evaluated by testing *in vitro* cultures of human HaCaT keratinocytes, a cell line immortalized from a 62-year old Caucasian male donor [23]. The cell type and the age of the original donor are crucial in the context of the present work, since hypoxia-associated pathologies of dermal tissues are more frequent in the elderly. Moreover, the production of human keratinocyte matrix metalloproteinases, a family of enzymes playing a key role in tissue repair and wound healing mechanisms, was shown to be differentially altered by hypoxia, depending on donor’s age [40]. Interestingly, OLNDs were avidly internalized by HaCaT cells and did not result toxic for cells, both in normoxic and hypoxic conditions, eventually improving keratinocyte viability.

As a next step, the abilities of OLND, OLNB, and OSS liquid or gel formulations to release oxygen *in vitro* were comparatively evaluated. Intriguingly, OLNDs released larger amounts of oxygen for longer times than OLNBs and OSS. Of note, OLND gel formulation, specifically developed and standardized to allow OLND topical use, displayed lower oxygen levels but faster oxygen release ability than OLND liquid formulation. This appears to be a consequence of the preparation protocol for the gel formulation (see Materials and Methods), which might likely cause a slight loss of oxygen content and lead to the formation of aggregates in the emulsion, thus justifying both lower oxygen levels and faster release dynamics. Nevertheless, those alterations did not affect our purposes, since OLND formulation still appeared more effective than OLNB and OSS formulations. As previously discussed, while binding oxygen molecules, DPF can establish hydrogen bonds, unlike PFP. Therefore, OLND cores likely contain more oxygen than OLNBs, thus justifying why oxygen release from OLNDs resulted higher and longer-lasting.

Oxygen release was also compared following sonication. In fact US is expected to impact on oxygen release kinetics through several mechanisms. Firstly, US can induce bubble formation after acoustic droplet vaporization [41]. Under particular gas content and bubble radius conditions, bubble oscillations might lead to a more violent release mechanism due to cavitation, that is the formation, growth, and implosive collapse of bubbles in a liquid [42–43]. Finally, US might elicit sonophoresis: indeed, it has been proven that the cellular uptake of drugs and genes is increased when the region of interest is under US administration, especially in the presence of a contrast agent, and such an increased uptake has been attributed to the formation of transient porosities in the cell membrane, which are big enough for the transport of drugs into the cell [44–47]. For instance, preclinical and clinical evidence that combined sonoporation and chemotherapy effectively impede development of pancreatic cancer either in mice or in human patients has become available recently [48–49]. According to our results, US effectively improved the ability of OLNDs to release oxygen through a pig skin layer into a hypoxic chamber, with such oxygen release being larger than that from OFND, OLNB, OFNB, and OSS liquid or gel formulations.

OLNDs were finally tested *in vivo*. The skin oxygenation of mice topically treated with OLND, OFND or OSS gel formulations was monitored by visualizing the subcutaneous levels of oxy-Hb and deoxy-Hb through photoacoustic imaging. This innovative hybrid imaging technique, based on the light absorption and the acoustic transmission properties of a tissue slice interrogated by a computed tomography photoacoustic imager [50–51], can quantify the density of tissue chromophores such as oxy-Hb and deoxy-Hb, measuring physiological parameters such as blood oxygen saturation and total Hb concentration [52]. According to our results, oxy-Hb levels significantly increased for the entire observational period in the animals treated with OLNDs, whereas OSS induced a high but only transient peak in oxy-Hb and OFNDs did not affect oxy-/deoxy-Hb balances at all. Of note, the fluence level employed for the present photoacoustic measurements were below 20 mJ/cm^2^, in accordance with the ANSI standard of maximum permissible exposure limit to the skin [53]. Therefore, the laser used in the photoacoustic system did not cause droplet evaporation, which generally occurs at very high fluence. For example, fluence values for water droplets are ~3 J/cm^2^ independent of drop size [54].

OLND ability to improve tissue oxygenation *in vivo* was also investigated upon US treatment. Transcutaneous oxymetry was chosen to perform this analysis since it measures the oxygen transcutaneous tension (*tcpO*_*2*_) through a non-invasive method which elicits a heating-related vasodilatation, generating fast diffusion of gases from the vessels to an electrode located on the skin. When capillary oxy-Hb dissociation occurs, the reaction of oxygen reduction generates a current which is directly proportional to capillary oxygen arterial pressure. Monitoring *tcpO*_*2*_ is a well-consolidated technique extensively used also in clinical practice [2, 55]. Basal *tcpO*_*2*_ values in mice were inhomogeneous, possibly as a consequence of the different level of peripheral vasoconstriction induced by anesthesia [38]. Nevertheless, after topical administration of US-activated OLNDs, hypoxic mice displayed larger oxygenation levels in a time-sustained manner. Interestingly, US-activated OLNDs did not alter basal *tcp*O2 in those mice which were already normoxic *per se*. This appears particularly relevant, since hyperoxia might eventually lead to alterations of cardiac, vascular, and respiratory functions as well as developmental disorders [56–57].

In conclusion, the DFP-based oxygen nanocarriers described here appear as innovative, promising, nontoxic, and cost-effective therapeutic tools for hypoxia-associated dermal pathologies. Dextran-shelled OLNDs, with 600 nm average diameters, negative charge, good oxygen capacity, and without toxic effects on human keratinocytes, can be manufactured both in liquid and gel formulations, being the latter more suitable for topical administration. OLNDs have higher effectiveness in releasing oxygen to hypoxic media and superficial tissues compared to OLNBs and OSS. Sonication further enhances transdermal oxygen delivery from OLNDs, possibly by promoting cavitation and sonophoresis. Therefore, future preclinical and clinical studies look encouraging as OLNDs offer promising treatment of chronic wounds, including bedsores, critical limb ischemia and diabetic foot.
